# 

**Published:** 1906-04-21

**Authors:** 


### Medical Appointments and Vacancies.

### APPOINTMENTS.

Jones, H. Travers, M.R.C.S., L.R.C.P., Resident Medical Officer to the Hospital for Epilepsy and Paralysis, Maida Vale.

### VACANCIES.

Birkenhead Borough Hospital.—Junior Resident House Surgeon.
Birmingham, University of.—Assistant Lecturer on Pathology and Bacteriology.
Bodmin, Cornwall County Asylum.—Third Assistant Medical Officer.
British Lying-in Hospital.—Physician.
Central London Throat and Ear Hospital, Gray's Inn Road, W.C.—Third Registrar (honorary).
Essex and Colchester General Hospital.—Honorary Assistant Surgeon. Also House Surgeon.
Evelina Hospital for Sick Children, Southwark, S.E.—Surgeon.
Fazakerley, City of Liverpool Infectious Diseases Hospital.—Assistant Resident Medical Officer.
Hellingly, East Sussex County Asylum.—Second Assistant Medical Officer.
Hemel Hempstead, West Herts Infirmary.—House Surgeon and Assistant Secretary.
Hospital for Sick Children, Great Ormond Street, London, W.C.—House Surgeon.
Huddersfield Infirmary.—Junior House Surgeon.
Leicester Poor-law Infirmary.—Second Resident Assistant Medical Officer.
Liscard, Wallasey Dispensary and Victoria Central Hospital.—House Surgeon.
London County Asylum, Colney Hatch, New Southgate, N.—Third Assistant Medical Officer.
Paddington Green Children's Hospital, London, W.—Second Honorary Anæsthetist.
Royal National Orthopædic Hospital, 234 Great Portland Street, W.—Resident House Surgeon.
St. Paul's Hospital for Skin and Genito-urinary Diseases, Red Lion Square, W.C.—Honorary Assistant Surgeon and two Casualty Out-patient Surgeons.
University College, London.—Resident Medical Officer.

In his annual report to the Corporation of the City of London the medical officer of health states that much attention has been given to the character of the milk supply to the City, and a further series of bacteriological analyses of milk samples from twenty-two different counties show unmistakably the great need for a stricter supervision of dairy farms. Having cited nine instances of the production of a warranty by defendants in prosecutions leading to the cases being dismissed, Dr. Collingridge affirms that unless some amendment or repeal of the existing warranty clauses of the Sale of Food and Drugs Act is made, adulteration, especially of milk, will flourish exceedingly.

### NOTICE TO CORRESPONDENTS.

All MSS., Letters, Books for Review, and other matters intended for the Editor should be addressed The Editor, "The Hospital," The Hospital Buildings, 28 & 29 Southampton Street, Strand, W.C.

The Editor cannot undertake to return rejected MS., even when accompanied by stamped directed envelope.

**Notice to Advertisers and Subscribers.**

All Advertisements, Orders for Copies of The Hospital, and Business Communications should be addressed to THE MANAGER (Not to the Editor), The Hospital, 28 & 29 Southampton Street, Strand London, W.C.

The Cost of Subscription, post free, is as follows :—Per week, 3½d. ; per quarter, 3s. 3d. ; per half-year, 6s. 6d. ; per annum, 13s. Foreign and Colonial :—Per annum, 19s. 6d., payable, in all cases, in advance.

**NOTICE.—Vol. XXXIX. of "The Hospital" (October 1905 to March 1906), in handsome cloth binding, will shortly be on sale at the office of this Journal, price 10s. 6d. Cases for binding the Numbers contained in this Volume 2s. Index to Vol. XXXIX., 3½d., post free.**

**The Monthly Part for April is now ready. Price 1s., by post, 1s. 3d.**

### IF YOU WANT A GOOD NURSING BOOK look for

### this Sign on the reverse of the title page. All our publications will in future bear this mark, and our aim is to produce just the Manual a Nurse requires to help her in the study of a new subject, or as a work of reference after the knowledge has been acquired; in fact, the best and most up-to-date Handbooks on Nursing and kindred subjects. You may be sure, therefore, that any book bearing this sign is the book on its subject. The large and increasing sale of our Publications, which are found in the hands of Nurses in all parts of the globe, is indisputable evidence of our success.

### These are some of our Books

**A HANDBOOK FOR NURSES. MEDICAL AND SURGICAL NURSING.**
By J. K. WATSON, M.D. New and Revised Edition.
Profusely Illustrated. **5s.** net, **5s. 4d.** post free.

**NURSING: its Theory and Practice.**
A Complete Text-book of Medical, Surgical, and Monthly Nursing.
By Dr. PERCY LEWIS. Profusely Illustrated. **3s. 6d.** post free.

**NURSING HINTS TO PROBATIONERS ON PRACTICAL WORK.**
By MARY ANNESLEY VOYSEY.
Illustrated. **2s.** net, **2s. 3d.** post free.
A complete Guide to the duties of a Nurse. An invaluable handbook to anyone desiring to enter the Nursing Profession.

**MEDICAL ELECTRICITY AND THE LIGHT TREATMENT.**
By Sister KATE NEALE, Light Department, Guy's Hospital.
Illustrated. **2s. 6d.** net, **2s. 9d.** post free.

**PRACTICAL GUIDE TO SURGICAL BANDAGING AND DRESSINGS.**
By WM. JOHNSON SMITH, F.R.C.S.
Profusely Illustrated. (Suitable size for apron pocket.) **2s.** post free.

**SURGICAL INSTRUMENTS AND APPLIANCES USED IN OPERATIONS.**
By HAROLD BURROWS, F.R.C.S. A Classified and Illustrated List with Explanatory Notes. **1s. 6d.** net, **1s. 8d.** post free.

**SURGICAL WARDWORK AND NURSING.**
By ALEXANDER MILES, M.D.
Illustrated. **3s. 6d.** net, **3s. 10d.** post free.

**LECTURES ON NURSING IN INFECTIOUS DISEASES.**
By Dr. J. WOOLLACOTT. Cloth, **2s. 6d.** net.
**The latest book on Fever Nursing.**

**A COMPLETE HANDBOOK OF MIDWIFERY.**
By J. K. WATSON, M.D.
350 pages, with 143 Illustrations. **6s.** net, **6s. 4d.** post free.
**The Handbook** for Candidates for the C.M.B. Exam.

**THE NURSE'S PRONOUNCING DICTIONARY OF MEDICAL TERMS AND NURSING TREATMENT.**
By HONNOR MORTEN. Cloth, **2s.** post free.
Fifth Edition, Edited and Revised by Mary I. Burdett.
Leather, **2s. 8d.** post free.

**SIMPLE SANITATION: the Practical Application of the Laws of Health to Small Dwellings.**
By M. LOANE. With an Introductory Chapter on Sanitary Legislation by Dr. A. Mearns Fraser, Medical Officer of Health, Portsmouth. **1s.** net, **1s. 2d.** post free.

**THE EXAMINATION OF THE URINE.**

By J. K. WATSON, M.D. **1s.** net, **1s. 1d.** post free.

*We shall be pleased to send you free of cost on receipt of postcard a copy of our Catalogue, which contains many other works on various Nursing subjects equally as good as the above books.*

### The Scientific Press, Ltd.,

***Catalogue of Nursing Manuals sent post free on application.***

**Publishers of Nursing Text-Books, Charts, and Case-Books of all Descriptions.**

**28 & 29 SOUTHAMPTON STREET, STRAND, LONDON, W.C.**

### THE UNIFORM SYSTEM OF ACCOUNTS

**For Hospitals and all Classes of Public Institutions.**

With Special Forms of Account and Specimen Rulings of a Complete Set of Books.

By SIR HENRY BURDETT, K.C.B.

New Edition. Royal 8vo. cloth, 4s. net; 4s. 4d. post free.

### ACCOUNT BOOKS FOR INSTITUTIONS

**Designed in accordance with the Uniform System of Accounts for Hospitals, &c.**

A Descriptive List of Books and Prices post free on application.

### THE INDEX OF CLASSIFICATION

Contained in "The Uniform System of Accounts," whereby every item of expenditure may be dealt with under identical heads by every group of Institution, is now published separately, price 1s.; 1s. 1d. post free.

### THE SCIENTIFIC PRESS, LTD.,

28 and 29 SOUTHAMPTON STREET, STRAND, LONDON, W.C.

